# The combinatory effect of scaffold topography and culture condition: an approach to nucleus pulposus tissue engineering

**DOI:** 10.2144/fsoa-2021-0157

**Published:** 2022-10-03

**Authors:** Noviana Vanawati, Anggraini Barlian, Hermawan Judawisastra, Indra Wibowo

**Affiliations:** 1 School of Life Sciences & Technology, Institut Teknologi Bandung, Bandung, West Java, 40132, Indonesia; 2 Faculty of Mechanical & Aerospace Engineering, Institut Teknologi Bandung, Bandung, West Java, 40132, Indonesia

**Keywords:** Human Wharton's jelly mesenchymal stem cells, hypoxia, low oxygen concentration, microenvironment, nucleus pulposus tissue engineering, platelet rich plasma, silk fibroin scaffold

## Abstract

Scaffold topography and culture medium conditions for human wharton's jelly mesenchymal stem cells (hWJ-MSC) are critical components of the approach to nucleus pulposus (NP) tissue engineering.

**Aim:**

To evaluate the silk fibroin (SF) scaffold topography analysis (optimal thickness and pore diameter) and to determine culture medium conditions for the growth and differentiation of hWJ-MSC.

**Method:**

hWJ-MSCs were seeded into different thicknesses and pore size diameters and grown in different concentrations of glucose, platelet rich plasma (PRP) and oxygen. The cell-seeded scaffold was evaluated for cell attachment, growth and differentiation potency.

**Results & discussion:**

The results indicated that SF scaffold with a minimum thickness 3.5 mm and pore diameter of 500 μm with cells cultured under low glucose, 10% PRP and normoxia conditions induced the growth and differentiation of hWJ-MSCs, indicated by the accumulation of glycosaminoglycans content and the presence of type II collagen, as markers of NP-like cells.

## Plain language summary

Until recently, the best approach to replacing a degenerating nucleus pulposus (NP) remained unclear. Tissue engineering is the most current method utilized to develop 3D cultures on scaffolds that direct cells sources into NP-like cell given optimal scaffold topography and culture conditions. Human Wharton's jelly mesenchymal stem cells (hWJ-MSC) are considered suitable multipotent stem cells for NP tissue engineering. A 3D construct of silk fibroin scaffold with a suitable thickness and pore diameter can facilitate attachment, growth and differentiation of hWJ-MSC into NP-like cells. Culture conditions with low glucose concentration on medium supplemented with PRP in normal oxygen conditions enhance the NP extracellular matrix marker, an indication that these 3D constructs and culture conditions can be developed into NP-like cells.

## Graphical abstract

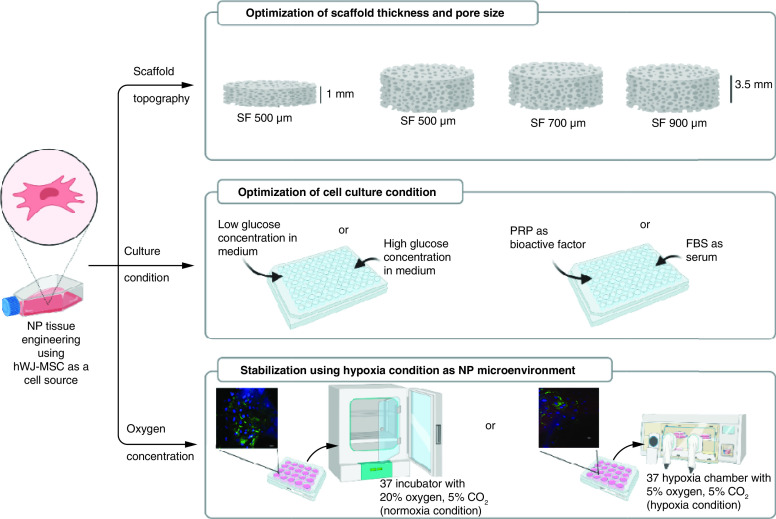

One of the causes of low back pain (LBP) is the degeneration of the intervertebral disc (IVD), which is composed of three distinct structures: annulus fibrosus, nucleus pulposus (NP) and cartilage end plate [1]. Given the NP's location in the IVD's deepest section, degeneration in the NP will harm the whole IVD. The therapy of NPs has been established around the use of mesenchymal stem cells (MSCs) as a source of cells to be directly transplanted into degraded NPs. However, cell-based therapy using transplanted MSCs into degraded NP can reduce MSC survival and function because cells will be exposed to harsh *in vivo* NP microenvironment conditions including low oxygen (hypoxia), low glucose, low pH and hyperosmolarity [2]. There is currently no conservative or surgical treatment that can effectively stop or slow the degenerative process in IVD, particularly in the NP section. Therefore, researchers developed a tissue engineering method to generate NP-like cells that can maintain their phenotype after implantation and restore degenerative NP to their original function [3].

Three critical components contribute to the success of NP tissue engineering: cells, scaffold materials and bioactive factors [4]. Human Wharton's jelly mesenchymal stem cells (hWJ-MSC) are a multipotent source of cells for tissue engineering since they can develop into chondrocytes, osteocytes, and adipocytes [5,6]. As a bioactive factor, 10% platelet-rich plasma (PRP) could promote proliferation and chondrogenic differentiation in MSC cultured [7]. Scaffold biomaterial as the third component, must fulfil two requirements i.e. biodegradable and biocompatibility. Silk fibroin (SF) as a biomaterial produced by *Bombyx morii*, has been demonstrated to meet those criteria [8]. SF scaffold is widely used in biomedical applications compared with synthetic materials. SF is mechanically strong and flexible, non-toxic, does not induce an immune response and is known to induce regeneration in cartilage tissue [9–11]. SF scaffold also shows structures that can facilitate cell activity such as proliferation, migration or differentiation, interconnected pores, good hydrophilicity and high water absorption ability [12]. According to previous, the mechanical strength of SF scaffolds is greater than that of scaffolds based on collagen, chitosan, HA and other biomaterial, making them more suitable for cartilage tissue repair [13]. At the same time, cell culture combination with low glucose and low oxygen condition, which is the characteristic of harsh microenvironment, promotes proliferation and facilitates better chondrocyte differentiation [14,15]. Liu *et al.* [16] demonstrated how low glucose circumstances influence NP cells *in vitro* and Naqvi & Buckley [17] showed the same effect on BMSC. Several studies have shown that hypoxia has a direct effect on inducing MSC differentiation by increasing type II collagen expression in 3D scaffold [18–20].

A previous study showed that a 500 μm pore size with 1 mm thickness is required for ADSC growth [21]. There has never been an investigation of the effect of scaffold topography in bigger pore size and thicker scaffold on cell proliferation and differentiation, considering the thickness of the NP varies between 7 and 12 mm. It is necessary to evaluate the influence of minimum scaffold thickness based on previous study [22]. The minimum standard for pore size and scaffold thickness was established in accordance with Barlian *et al.* [21] because it utilizes the same scaffold material, namely silk fibroin and the method for scaffold fabrication is based on Wibowo *et al.* [8]. SF scaffold can be formed in the shape of a 3D sponge, which is known to promote chondrocyte cell growth [23]. There has never been a study to determine whether pores larger than 500 μm influence cell proliferation and differentiation. Additionally, given the NP's thickness ranges between 7 and 12 mm in height, the scaffold must be made thicker for its application. Until now, little is known about the scaffold's effect on the proliferation of hWJ-MSCs *in vitro*. The thickness to be tested is the minimum thickness determined by multiplying the porogen's size by 3.5 to obtain a stable scaffold by previous research [22].

Preliminary research has demonstrated that ADSC cultured on SF scaffolds in medium containing 10% pletelet rich plasma (PRP) can promote growth and chondrogenesis [21]. TGF-β1 is one of the multiple growth factors found in PRP and it is involved in the formation of chondrocytes in ADSC [24]. However, additional research is required to discover whether the same culture conditions with different cells can enhance growth and differentiation and how additional PRP in hypoxic and normoxic environments is required for the formation of intervertebral disc chondrocytes. At the same time, cell culture condition with low glucose concentration and hypoxia which is the characteristic of harsh microenvironment, promotes proliferation and facilitates better chondrocyte differentiation in MSC [17,20]. The combination of culture medium with low glucose concentration and hypoxic conditions affects hWJ-MSC cultured on 3D SF scaffold and its involvement in NP tissue engineering with NP extracellular matrix (ECM) markers (Glycosaminoglycans and type II collagen) has not been identified.

The primary goal of this research was to analyze the effect of increasing the SF scaffold thickness to 3.5 mm and the SF scaffold pore size to 500, 700 and 900 μm on the proliferation and differentiation of hWJ-MSC. The secondary goal was to optimize the culture condition in the NP microenvironment, including PRP, low glucose concentrations in the medium and hypoxic conditions, to facilitate hWJ-MSC proliferation and differentiation into NP-like cells that express NP ECM markers.

## Materials & methods

### Fabrication of SF scaffold

The SF was obtained from *Bombyx mori* cocoons (CV. Wisata Ilmu Sutra, Bandung, West Java, Indonesia) and fabricated using a modified salt leaching technique for added thickness, as previously describe by Wibowo *et al.* [8]. To begin fabricating the porous silk fibroin (SF) scaffold, the silk cocoon was degummed twice with 0.5 wt% NaHCO to remove the sericin. Rinsed silk cocoon with demineralized water and then dried in a fume hood overnight. 12-wt% CaCl_2_-formic acid was used to dissolve the degummed silk and then mix in the 500, 700 or 900 μm sodium chloride (NaCl) as a porogen in 2.5 cm mold to create a scaffold. NaCl and dissolved silk were mixed at a ratio of 5:1. The solvent was removed from the NaCl-silk mixture by placing it in a fume hood overnight. After evaporating the solvent from the NaCl-silk mixture, the 3D SF scaffold was immersed in 70% ethanol for one hour, followed by 3 days for the dialysis process by demineralized water changed every 6 h to remove the salt. 3D SF scaffold was then cut into 5x5 mm size and sterilized at 121°C for 15 min before further examination.

### hWJ-MSC isolation & culture

The primary culture of hWJ-MSC was performed using an enzymatic solution based on a modification of the method used by Salehinejad *et al*. [25]. This method included the addition of 2% collagenase for 30 min followed by trypsin (Gibco) for 16–18 h in an incubator at 37°C. When Wharton's jelly formed, it was dissolved in high glucose growth medium Dulbeccos's Modified Eagles' Medium (DMEM, Gibco) supplemented with 10% fetal bovine serum (FBS, Gibco) and 1% antibiotic-antimycotic (Gibco) as culture standard medium. The medium was changed every 2 days and the culture was incubated at 37°C with 5% CO_2_. After 12–14 days of incubation, the primary cells adhered to the substrate and grow until they have reached 80% confluency (Passage 0, P0). The primary cells were examined further after passage 5.

### Characterization of hWJ-MSC

hWJ-MSC were characterized to confirm that cells fulfilled International Society for Cellular Therapy (ISCT) requirements for MSC, which include cell attachment, multipotency assay and MSC-specific surface marker. Attachment cells analysis. 10^4^ hWJ-MSC were cultured on 24-well plate until 80% confluence and then imaged using an inverted microscope.

Multipotency assay. 10^4^ hWJ-MSC were cultured on 24-well plates and incubated in culture standard medium. After 80% cells confluency, the medium was replaced with chondrogenic medium (StemPro™ chondrogenesis differentiation kit), osteogenic medium (StemPro™ osteogenesis differentiation kit) and adipogenic medium (StemPro™ adipogenesis differentiation kit). After 21-day incubation, cells were fixed with 4% paraformaldehyde and stained with Alcian blue to detect glycosaminoglycans (GAGs) accumulation, Alizarin red to detect calcium deposits and Oil red O to detect lipid droplets. Following that, the staining results were evaluated using an inverted microscope.

MSC specific surface marker. 3 × 10^5^ hWJ-MSC were cultured in T-25 flasks and incubated in culture standard medium until 90% confluency. After a trypsinization procedure, cells were counted to 10^6^ cells in 1 ml of buffer. Every 10^5^ cells were divided into 3 microtubes and then tested using MSC Analysis Kit (BD Bioscience) which contained specific mesenchymal surface marker. Each sample received 500 μl of positive cocktail (CD90 FITC, CD105 PerCP and CD73APC), negative cocktail (CD34, CD 11, CD19, CD45 and HLS-DR), or isotype. After 30 min of incubation in the dark, the samples were rinsed with staining buffer and 500 μl of buffer was added. The DB Accuri™ C6 cytometer was used to analyze flow cytometry data.

### Scaffold characterization

Scanning electron microscope (SEM) analysis of the SF scaffold was used to investigate the formation of pore and interconnected pore size in the scaffold. The scaffold was immersed in a 70–100% alcohol solution and then dried overnight using a Hexamethyldisilazane (HMDS, Electron Microscopy Sciences) solution. Samples were coated with gold and examined under SEM (SU 3500; Hitachi, Krefeld, Germany, Center of Advanced Science ITB). The ImageJ software was used to calculate the average pore size using three microscope areas and more than 40 different pore size different areas.

Water uptake measurement was performed to determine the SF scaffold's ability to absorb water for 24 h. The dry weight before immersion (W_0_) and the wet weight after immersion (W_1_) of the SF scaffold were determined using the following formula [8]:

$$\mathrm{Water uptake}=(W_{1}-W_{0})/W_{0}\times 100\%$$

Compressive testing was performed to determine SF scaffold's resistance to compressive stresses using TENSILON Universal Testing Machine. The machine compressed the scaffold approximately 50% strain at compressive speed of 0.5 mm/min [26].

### SEM analysis of hWJ-MSC morphology grown on a SF scaffold

10^5^ hWJ-MSC grown on a 3D SF scaffold for 72 h. The samples were fixed with 2.5% glutaraldehyde in 0.1 M cacodylate buffer for 2 h at room temperature, then dehydrated with a series of alcohols ranging from 30% to 100% and dried overnight with Hexamethyldisilazane (HMDS, Electron Microscopy Sciences). Samples were plated with gold and observed using a SEM (SU 3500; Hitachi, Krefeld, Germany, Center of Advanced Science ITB).

### hWJ-MSC culture & seeding

hWJ-MSC passage 5 were utilized in all treatments with three replicates of each analysis. For evaluating cell growth and differentiation potency, 2.5 × 10^4^ hWJ-MSC were seeded on the SF scaffold for 2 thickness variation (1 mm and 3.5 mm) and 3 pore sizes variations (500, 700 and 900 μm). Hypoxia conditions were analyzed using a hypoxia chamber containing 5% O_2_, while normoxia conditions were analyzed using a cell culture incubator containing 20% O_2_.

For optimize the medium's glucose concentration and PRP concentration, 10^4^ hWJ-MSC were seeded in the monolayer seeding treatment without scaffold. Each cell was treated in a specific medium containing either low glucose (1 g/l) or high glucose (4.5 g/l) DMEM medium to optimize the medium's glucose concentration and 5, 10 or 20% PRP to optimize the PRP concentration.

### Growth & differentiation potency analysis of hWJ-MSC

Growth analysis was performed using the (MTT) Methylthiazolyldiphenyl-tetrazolium bromide, Sigma Aldrich cytotoxic assay. Cell growth was determined solely by the increase in the absorbance value of dissolved formazan crystal that had formed from live cells. Increased absorbance indicates an increase in the number of cells from day one. The accumulation GAGs in the extracellular matrix was analyzed as a sign of chondrogenesis based on previous method [7]. For 3 min, the scaffold and cells were incubated in a solution of methanol and acetone (1:1) at 4°C. Afterward, the samples were incubated in 1% Alcian blue for 3 min. Following that, rinsing the sample with 3% acetic acid and then with distilled water to eliminate any leftover residue. The samples were then incubated for 30 min with 1% SDS. GAGs content analysis was done by using spectrophotometry at wavelengths of 605 nm. Absorbance was found to be directly related to GAGs levels.

### The percentage of hWJ-MSC attached to SF scaffold

The counting of cells that attach to the scaffold is not easy because of the 3D structure. Therefore, the calculation of cell attachment using indirect methods is based on Valipour *et al.* [27] The cells in the pore were determined by whether the cells attach to the scaffold or whether they attach to the bottom of the well plate. After 24 h of incubation, cells that passed through the scaffold and attached to the bottom of well plates were counted by trypsinization and stained with trypan blue. The calculation is done by determining the percentage of cells attached by using the formula: initial number of seeded cells minus attached cells to the bottom of the well plate divided by the initial number of seeded cells (25,000 cells).

### Pore filling & cell penetration analysis by immunocytochemistry

Pore-filling examinations were carried out after 1, 3 and 7 days of incubation. Meanwhile, cell penetration was carried out after 21 days of incubation 3D cell-scaffold cultures were fixative with 4% paraformaldehyde for 30 min at room temperature before being permeabilization for 5 min with 0.1% Triton-X. Staining actin filament using 0.1% Rhodamine phalloidin (Invitrogen, AB235138) in 3% BSA-PBS for 90 min was followed by staining cell nuclei with 2.5 g/ml DAPI for 10 min in the dark. Observation was done with the use of a confocal microscope (Olympus Fv1200) and the Fluoview software.

### Type II collagen expression with immunocytochemistry

The type II collagen examination in hypoxia and normoxia conditions was carried out after 21 days of incubation. Construction of 3D cell-scaffold cultures was fixed by adding DMEM – methanol series (50% - 100%) for 5 min at -20°C as a fixative solution, followed by permeabilization with 0.05% Tween-20 in PBS for 5 min and blocking with 3% BSA for 5 min. Type II collagen (ab 34712, Abcam) was used as a primary antibody and Alexa Fluor 488 as a secondary antibody. 2.5 g/ml DAPI was added to stain the cell nucleus and incubated for 10 min in the dark. Observations were performed using a confocal microscope (Olympus Fv1200) and the Fluoview software.

### Western blotting analysis

After 7 days in normoxia or hypoxia conditions, cells on the scaffold were washed three-times with ice-cold PBS and total proteins were extracted using RIPA buffer. The Bradford method was used to determine the protein concentrations. Proteins were separated using 10% sodium dodecyl sulfate polyacrylamide gel electrophoresis (SDS-PAGE) and then transferred to PVDF membranes (Millipore, MA, USA). After transferring the proteins to the membrane, immunoblotting was performed using primary antibodies Col2, hif1-α, and Sox9 (Abcam) and a biotinylated secondary antibody anti-rabbit IgG (Abcam, ab 6740) as a secondary antibody (Roche 11520709001). The ECL (Enhanced Chemiluminescence, Roche 11520709001) system was used to detect proteins and visualize proteins for quantification using C-Digit (Liqor).

### Statistical analysis

Two-way analysis of variance (ANOVA) was performed to determine the significance of hWJ-MSC growth and GAGs content. Tukey's multiple comparison test was used for post-hoc analysis. All calculations for significance analysis were carried out using GraphPad prisms version 8.4.2 for Windows (GraphPad Software, CA, USA).

## Results

### hWJ-MSC characteristics

**Figure 1.**
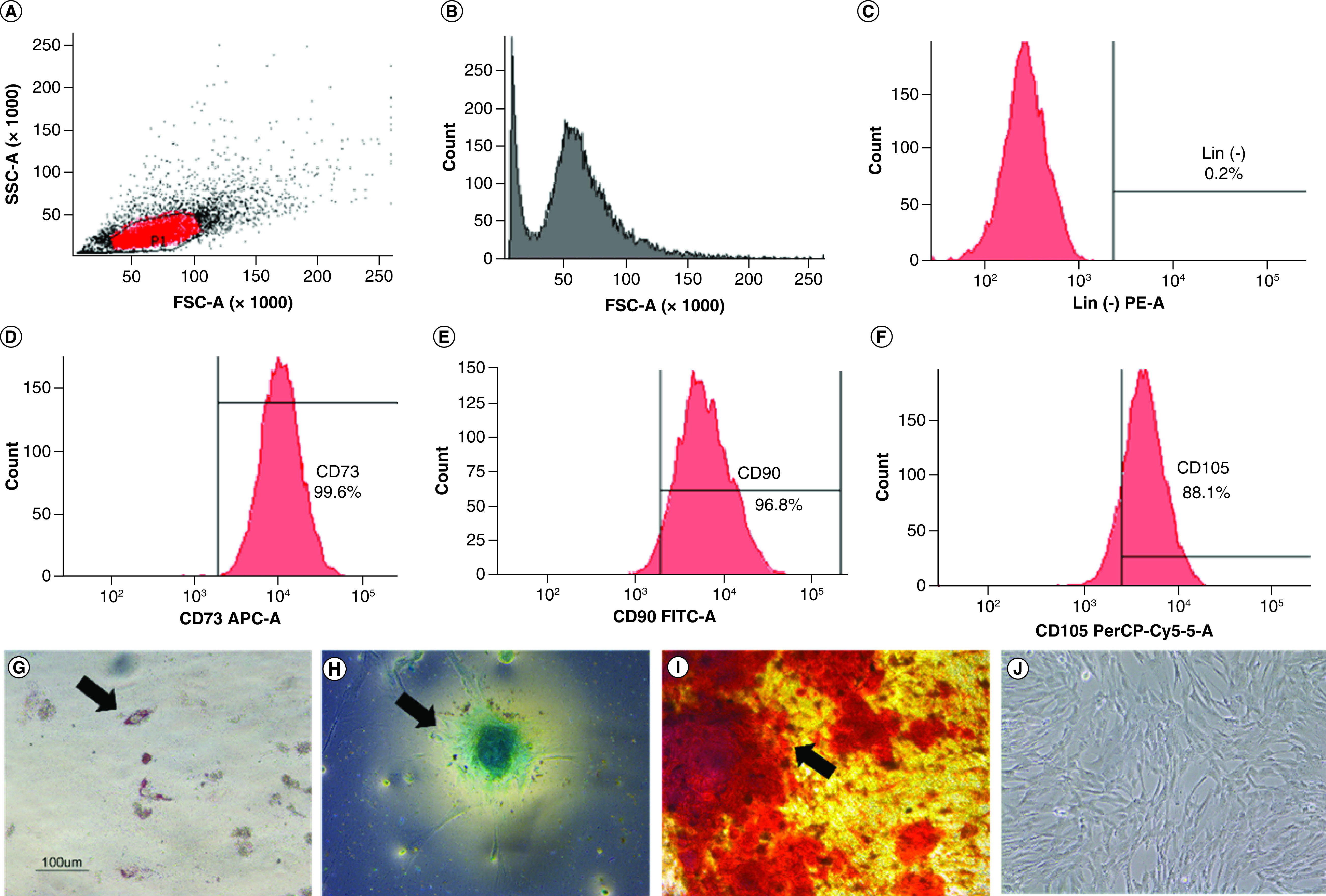
Characteristics of hWJ-MSC. Analysis of specific cell surface marker: **(A)** Plotting cells; **(B)** Negative isotype control. **(C)** Negative marker: CD45, CD34, CD11b, CD19, HLA-DR. Positive marker: **(D)** CD73; **(E)** CD 90; **(F)** CD 105. Multipotency analysis: **(G)** Adipocyte differentiation using Oil Red O, **(H)** chondrogenesis differentiation with Alcian blue staining and **(I)** osteocyte differentiation with Alizarin red staining. **(J)** hWJ-MSC morphology at 90% confluency. Black arrow shows positive result of differentiation. hWJ-MSC: Human Wharton jelly’s mesenchymal stem cells. Scale bar = 100 μm.

The characterization of hWJ-MSC was performed to confirm that the isolated cells fulfilled the International Society for Cellular Therapy (ISCT) requirements for MSC. Negative markers were present in less than 2% of the cells (Figure 1C), whereas positive markers (CD 73, CD 90, CD105) were present in more than 90% of the cells (Figure 1D–F). Multipotency analysis revealed that the cells could differentiate into adipocytes as evidence by fat droplets stained with oil red O (Figure 1G), chondrocytes as evidenced by GAGs accumulation using Alcian blue staining (Figure 1H) and osteocytes as evidenced by mineralization using Alizarin red staining (Figure 1I). Cell attachment on the substrate was proven in Figure 1J. Based on all these findings, the characteristic results fulfilled the ISCT requirement, and the isolated cells were hWJ-MSC [28].

### Optimization of SF scaffold thickness

**Figure 2.**
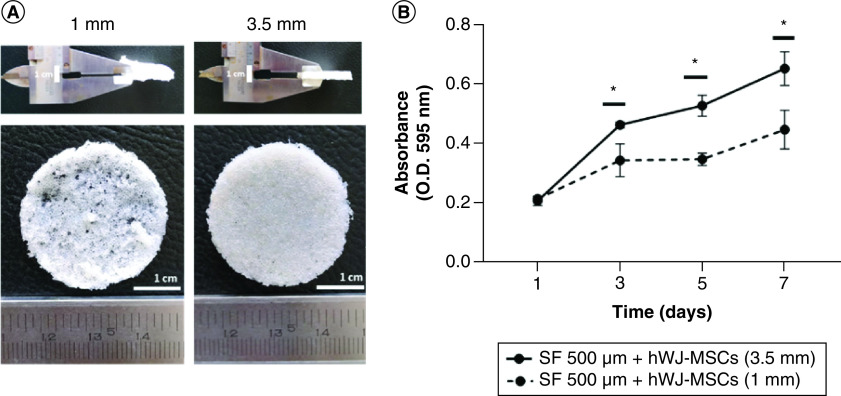
Optimization of scaffold thickness on the growth of hWJ-MSC. **(A)** General observation of SF scaffold. **(B)** The effect of SF scaffold thickness on the growth of hWJ-MSC. hWJ-MSC: Human Wharton jelly’s mesenchymal stem cells; OD: Optical density. *p = 0.0322. Scale bar: 1 cm. n: 3.

A previous study showed that when compared with other pore size, an SF scaffold with 500 μm pore size and a 1 mm thickness supported ADSC growth [21]. SF scaffold was made by the salt leaching method which has a sponge-shaped scaffold structure with different thickness (Figure 2A). Here, we modified the thickness of SF scaffold to 3.5 mm. As presented in Figure 2B, growth capacity of hWJ-MSC grown on scaffold with a 3.5 mm thickness was significantly higher than cells cultured on scaffold with a 1 mm thickness from day 1 to 7. The same cell seeding for different thickness is to ensure same variable for demonstrate that the minimum pore size for scaffold must be reached with the minimum scaffold thickness based on Ramadhianti *et al.* [22] research. This study shows that a certain thickness must be reached for optimal cell growth. Thus, SF scaffold with a thickness of 3.5 mm can be used in the following experimental study.

### SF scaffold characteristics with pore size variation

Regarding to SEM analysis, pores and interconnected pores formed in all variants of the scaffold, regardless of the size of the NaCl used as a porogen (Table 1 & Supplementary 1). The bigger the size of NaCl, the more porous and interconnected pores formed on the scaffold [29]. Water uptake analysis shows that the bigger the pores in the SF scaffold, the higher the effective to absorb the surrounding media, within 1 h (Table 2).

**Table 1.** Measurement of pores and interconnected pores with different sizes of NaCl (n = 3; mean ± SEM).

Pore name	Salt size	Scaffold pore size	Interconnected pore size
SF 500 μm	500–595 μm	407 ± 67 μm	71 ± 26 μm
SF 700 μm	675–750 μm	671 ± 86 μm	110 ± 47 μm
SF 900 μm	850–1000 μm	817 ± 89 μm	206 ± 106 μm

**Table 2.** Water content analysis (n = 3; mean ± SEM).

Water content (%)			
Observation time	SF 500 μm	SF 700 μm	SF 900 μm
1 h	61.4778 ± 1.49	77.24 ± 2.72	77.07 ± 2.5

In constructing the scaffold, it is necessary to consider its capacity to endure stress following implantation *in vivo* conditions. Compressive strength was used to determine the resistance of the SF scaffold with various pore sizes when exposed to a compressive force (Table 3). Pore size of 500 μm had the highest compression modulus and strength values compared with other pore sizes.

**Table 3.** Compression test result (n = 3; mean ± SEM).

Analysis	SF 500 μm	SF 700 μm	SF 900 μm
Compression modulus (MPa)	14 × 10^-3^ ± 0.47 × 10^-3^	7.7 × 10^-3^ ± 1.46 × 10^-3^	9.2 × 10^-3^ ± 0.98 × 10^-3^
Compression strength (MPa)	4.2 × 10^-3^ ± 0.23 × 10^-3^	2.2 × 10^-3^ ± 0.72 × 10^-3^	2.7 × 10^-3^ ± 0.23 × 10^-3^

### Effect of SF pore size on biocompatibility, percentage of cell attachment, growth & differentiation analysis of hWJ-MSC

**Figure 3.**
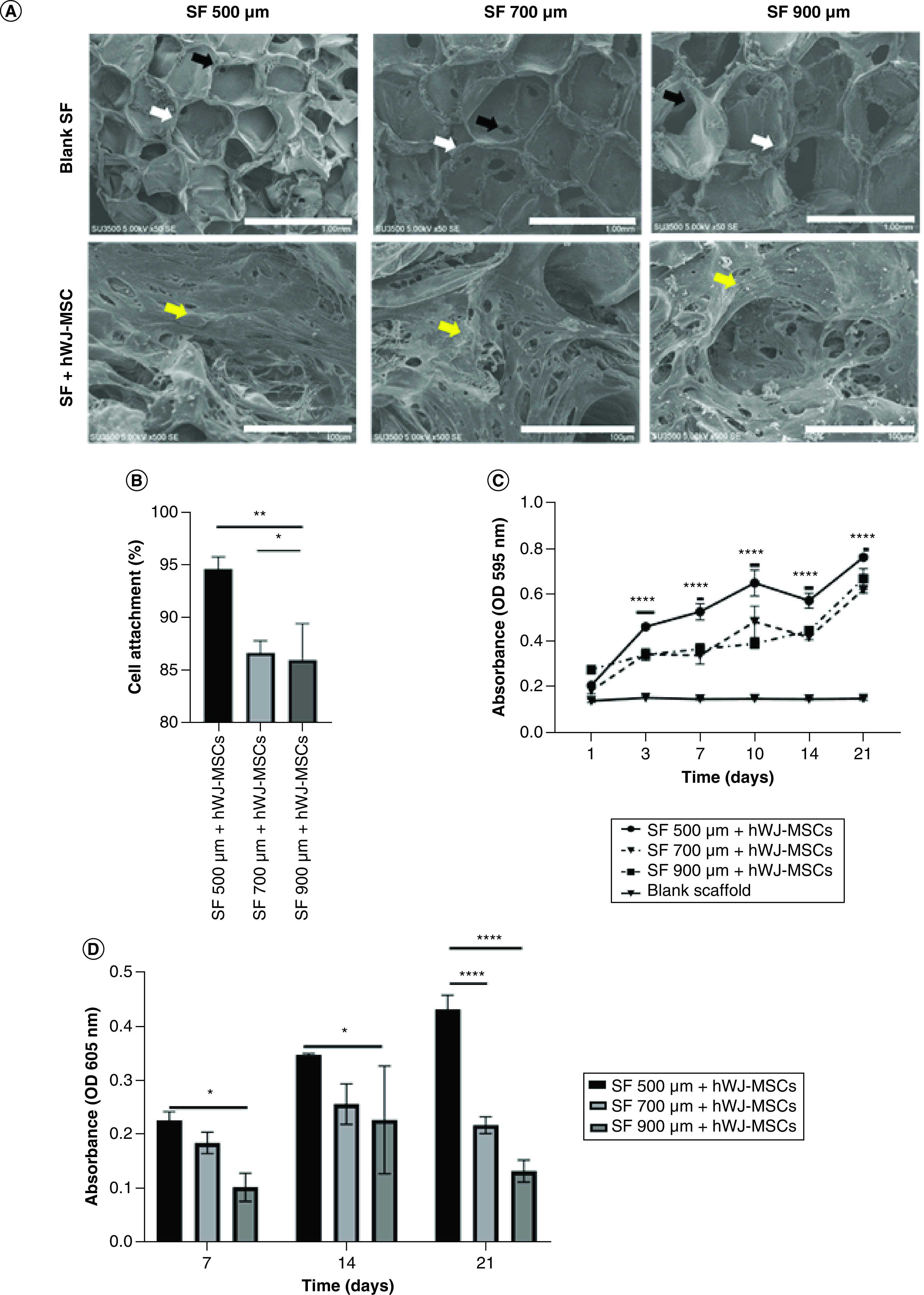
Analysis of hWJ-MSCs grown on SF scaffold with various pores. **(A)** SEM observation of SF pore structure and cell morphology attached on SF. **(B)** Percentage of hWJ-MSC attached to SF scaffold. **(C)** Growth curve of hWJ-MSC. **(D)** Graph of GAGs content. White arrows indicate form of pore, black arrow indicates interconnected pore, while yellow arrow indicates cells attachment. hWJ-MSC: Human Wharton jelly’s mesenchymal stem cells. *p: 0.0322; **p: 0.002; ***p: 0.002; ****p: <0.0001; n: 3.

In SEM analysis of blank scaffold (Figure 3A), different pores and interconnected pores formed in all variants of pore sizes depending on the amount of NaCl. The biocompatibility analysis of hWJ-MSC grown on SF scaffold indicated that cells were able to attach to the SF scaffold by showing the formation of a cell population (Figure 3A) in all the SF scaffold's pore variations. The percentage of cell attachment was evaluated to determine if pore size had an effect during initial cell seeding. This experiment shows that, 500 μm pore size scaffold has greater percentage of cells attach to the scaffold (Figure 3B), the highest growth curve (Figure 3C) and accumulation of GAGs content as differentiation marker until day 21 (Figure 3D).

### Analysis of pore filling & cell penetration of hWJ-MSC grown on SF scaffold with variable pore size

**Figure 4.**
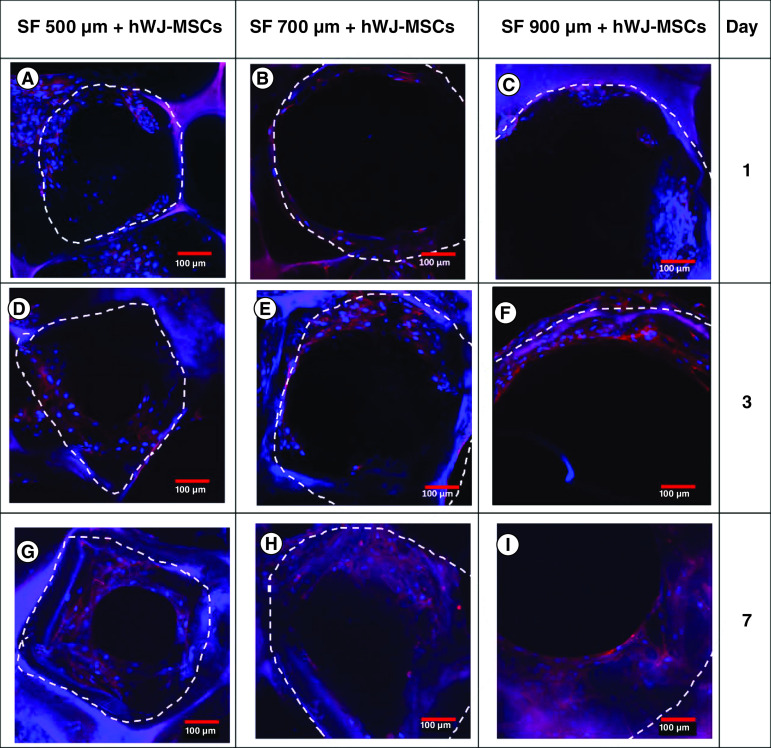
Pore filling trend of human Wharton jelly’s mesenchymal stem cells grown in a variation of pore diameters (3.5 mm scaffold thickness) as a function of incubation days. Confocal microscopy images of actin (red) and DAPI (blue) staining demonstrating the filling of SF scaffold pores by hWJ-MSC on: **(A–C)** day 1, **(D–F)** day 3, **(G–I)** and day 7, **(A,D & G)** with pore size variation of 500 μm; **(B, E & H)** 700 μm; **(C,F & I)** and 900 μm . The white dotted line marks the borders of scaffold porous structure. hWJ-MSC: Human Wharton jelly’s mesenchymal stem cells. Scale bar = 100 μm.

**Figure 5.**
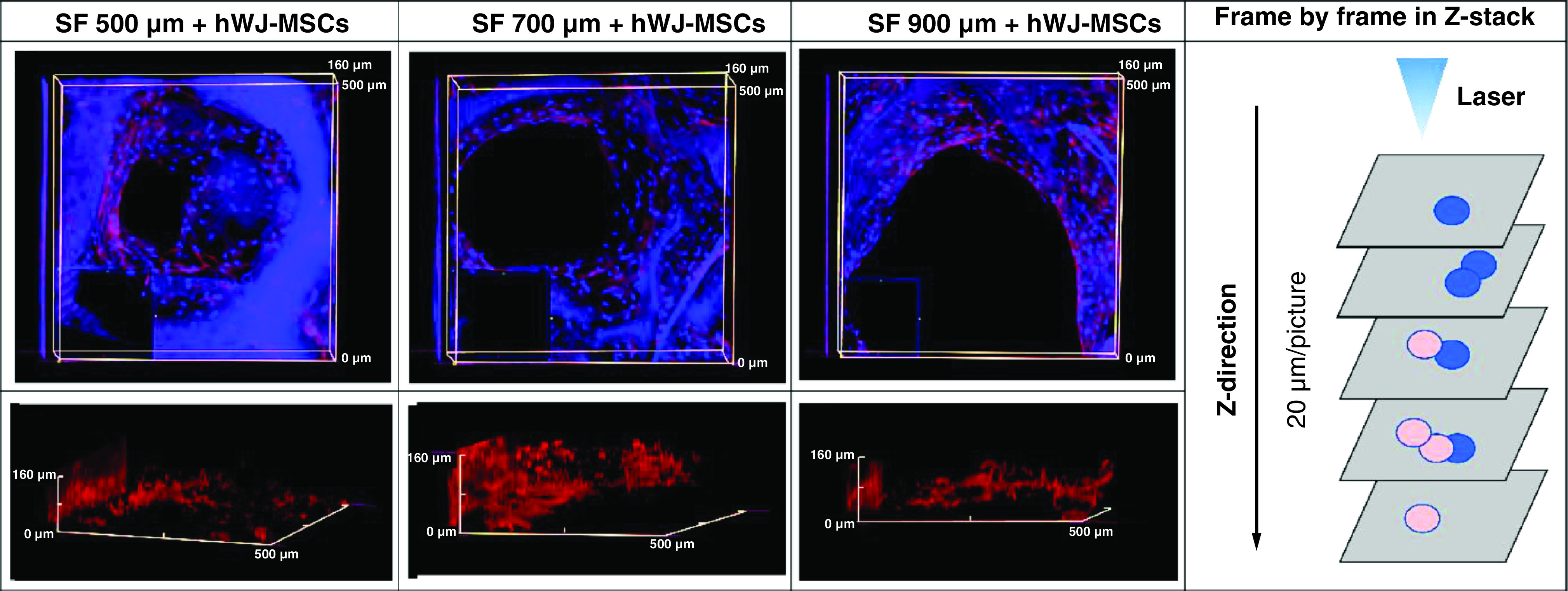
Confocal microscopy visualization of human Wharton jelly’s mesenchymal stem cells penetration into SF scaffolds with varying pore diameters (thickness of scaffold 3.5 mm). Confocal microscopy images of actin (red) and DAPI (blue) staining showing the 3D filling of SF scaffold pores by hWJ-MSC on day 21 with pore size variations of **(A & D)** 500 μm; **(B & E)** 700 μm; and **(C & F)** 900 μm. **(G)** Separated compartments cell and scaffold 3D image of Z-stack. **(A, B & C)** The photograph is taken from the pore's top to the **(D, E & F)** pore's side. The findings were captured using a z-stack at a depth of 20 μm each image. hWJ-MSC: Human Wharton jelly’s mesenchymal stem cells.

To prove the importance SF scaffold geometry determined by pore size directly impacts the proliferation, we observed the time required for cells to fill the pore region using immunocytochemistry (Figure 4). In 7 days of incubation, cells grown on a 500 μm SF scaffold were observed to spread across the pore more rapidly than cells grown on a larger pore size. Using z stack analysis on days 21 (Figure 5A–C), all the pores had closed and the space had been entirely filled with hWJ-MSC. Cells were able to penetrate through the 3D scaffold construction of the SF scaffold in all variants of the SF scaffold's pores, as determined by 3D observations (Figure 5D–F). Thus, the SF scaffold topography with a pore size of 500 μm and a thickness of 3.5 mm capable of supporting hWJ-MSC growth and differentiation and will be used in the following method. This result is corroborated by the compression strength result obtained with a 500 μm pore size of SF scaffold, which is significantly greater than the other pore size.

### hWJ-MSC growth & differentiation ability on a 500 μm SF scaffold with various cell culture media compositions

**Figure 6.**
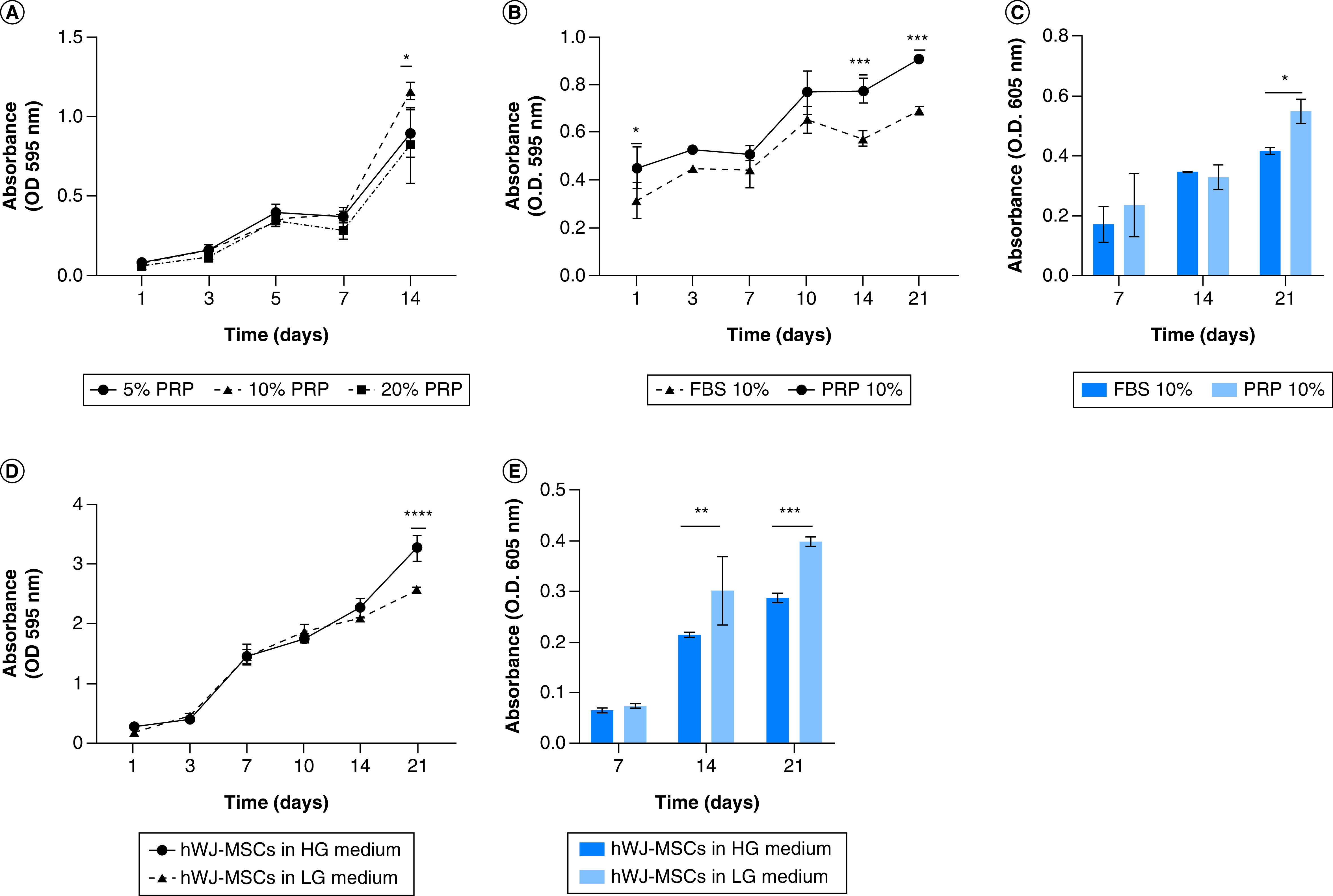
Growth curve and differentiation capability of human Wharton jelly’s mesenchymal stem cells for glucose and platelet rich plasma optimization. **(A)** Optimization of hWJ-MSC growth by PRP induction at various concentration without scaffold. **(B)** Growth curve effect of 10% PRP and 10% FBS induction on the growth of hWJ-MSC grown on scaffold and **(C)** the graph of GAGs content. **(D)** Effect medium low glucose and high glucose on growth of hWJ-MSC and **(E)** the GAGs content. *p: 0.0322; **p: 0.002; ***p: 0.002; ****p: <0.0001; n: 3. FBS: Fetal bovine serum; GAG: Glycosaminoglycan; hWJ-MSC: Human Wharton jelly’s mesenchymal stem cells; PRP: Platelet rich plasma.

Optimization of PRP revealed that a 10% PRP concentration was the optimal concentration for increasing hWJ-MSC growth at day 21 (Figure 6A). Based on these results, we compared the development of hWJ-MSC using 10% PRP to 10% FBS as a standard serum in culture medium (Figure 6B). The results indicated that in hWJ-MSC culture medium supplemented with 10% PRP, growth significantly higher at days 14 and 21, supported by measurement of GAGs content accumulation (Figure 6C). These findings are similar to work on hADSCs form previous research on hADSC [21].

Figure 6D show that on day 21, there was a significant increase in growth of hWJ-MSC grown on high glucose medium compared with low glucose medium. In contrast to the chondrogenic differentiation capability, hWJ-MSCs cultured in low glucose medium on days 14 to 21 show significantly higher accumulation of GAGs as a differentiation marker than those cultured in high glucose medium (Figure 6E), as shown by previous study [30].

### hWJ-MSC growth & differentiation ability on a 500 μm SF scaffold under 5% hypoxia conditions

**Figure 7.**
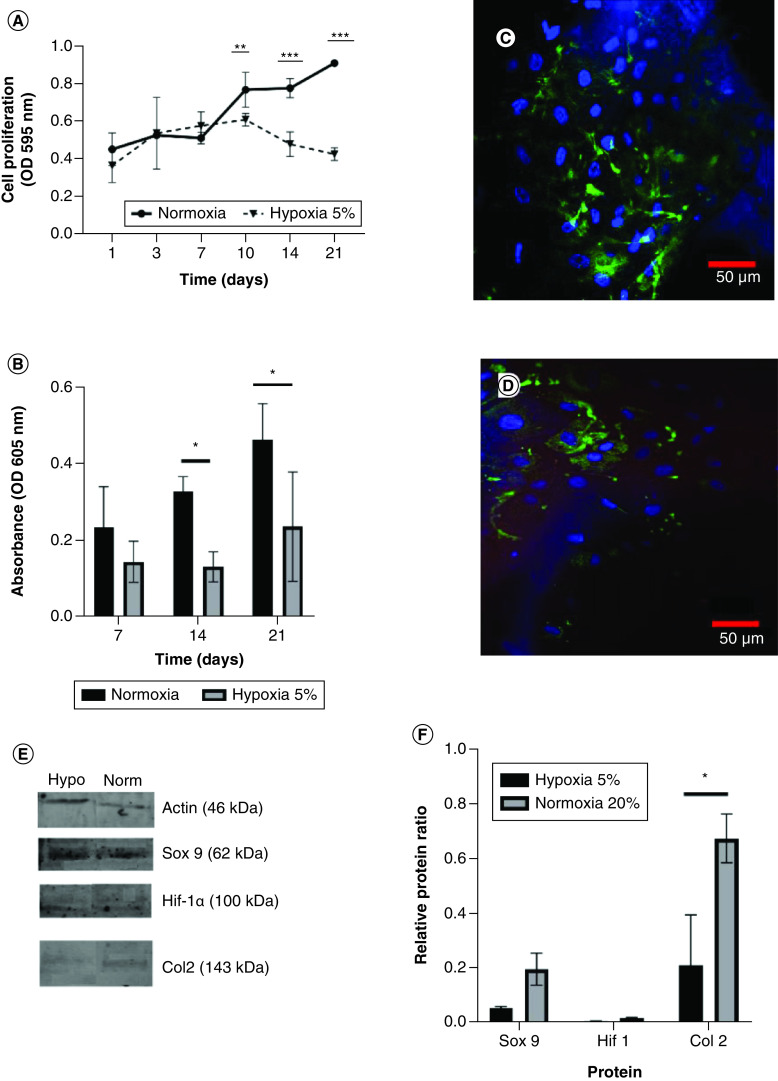
The effect of 5% hypoxia on the growth of human Wharton jelly’s mesenchymal stem cells implanted on a 500 μm pore size scaffold in the presence of a low glucose medium and 10% PRP induction, compared with normoxia condition. **(A)** Growth curve of human Wharton jelly’s mesenchymal stem cells in different oxygen concentration and **(B)** graph of GAGs content. **(C)** Confocal microscopy visualization of type II collagen expression in hWJ-MSC for 21 days under normoxia and **(D)** under 5% hypoxia condition. **(E)** Sox9, Col2, and Hif-1α expression by western blotting. **(F)** Relative ratio protein. Blue color indicates collagen type II collagen staining and blue color indicates DAPI staining. *p: 0.0322; **p: 0.002; ***p: 0.002; ****p: <0.0001; n: 3. hWJ-MSC: Human Wharton jelly’s mesenchymal stem cells.

Analysis of hypoxia conditions demonstrates no difference in the growth curves of hWJ-MSC cultured in 5% hypoxia conditions until day 21. However, there is an increase in the growth of hWJ-MSC cultured in normoxia from day 7 to day 21 (Figure 7A). This finding is corroborated by the differentiation potential graph (Figure 7B). Confocal microscopy visualization of hWJ-MSC (indicated by blue DAPI staining) and type II collagen expression as a marker of chondrogenesis were significantly greater in normoxia (Figure 7C) than in 5% hypoxia condition (Figure 7D). This result is supported by Western blotting analysis in Figure 7E & F.

## Discussion

The purpose of this work was to analyze optimum combination of human Wharton jelly's MSC (hWJ-MSC), Silk Fibroin (SF) scaffold topography and culture conditions for generating NP-like cells. Silkworm cocoon (*Bombyx mori*) as the main scaffold material of SF scaffold was degummed to remove sericin protein, which can induce an inflammatory response [31]. Scaffold fabrication based on the direct salt leaching technique, which involves dissolving 12 w/v CaCl_2_ / formic acid and NaCl as a porogen agent in water. This technique leads to a 3D scaffold with an amorphous α-helix and a crystalline β-sheet, secondary fibroin structure seen in SF [8].

The findings of the MSC characteristics analysis revealed the morphology and expression of cell surface antigens extracted from primary cultures utilized in the study using International Society for Cellular Therapy (ISCT) standard criteria [28]. The multipotency test findings established that hWJ-MSCs can develop into chondrocytes, adipocytes and osteocytes. This result is consistent with Mallis *et al.* finding that hWJ-MSC exhibits multipotency when grown in induction media [32]. Therefore, the cell employed in this investigation as a source of cells was hWJ-MSC.

**Figure 8.**
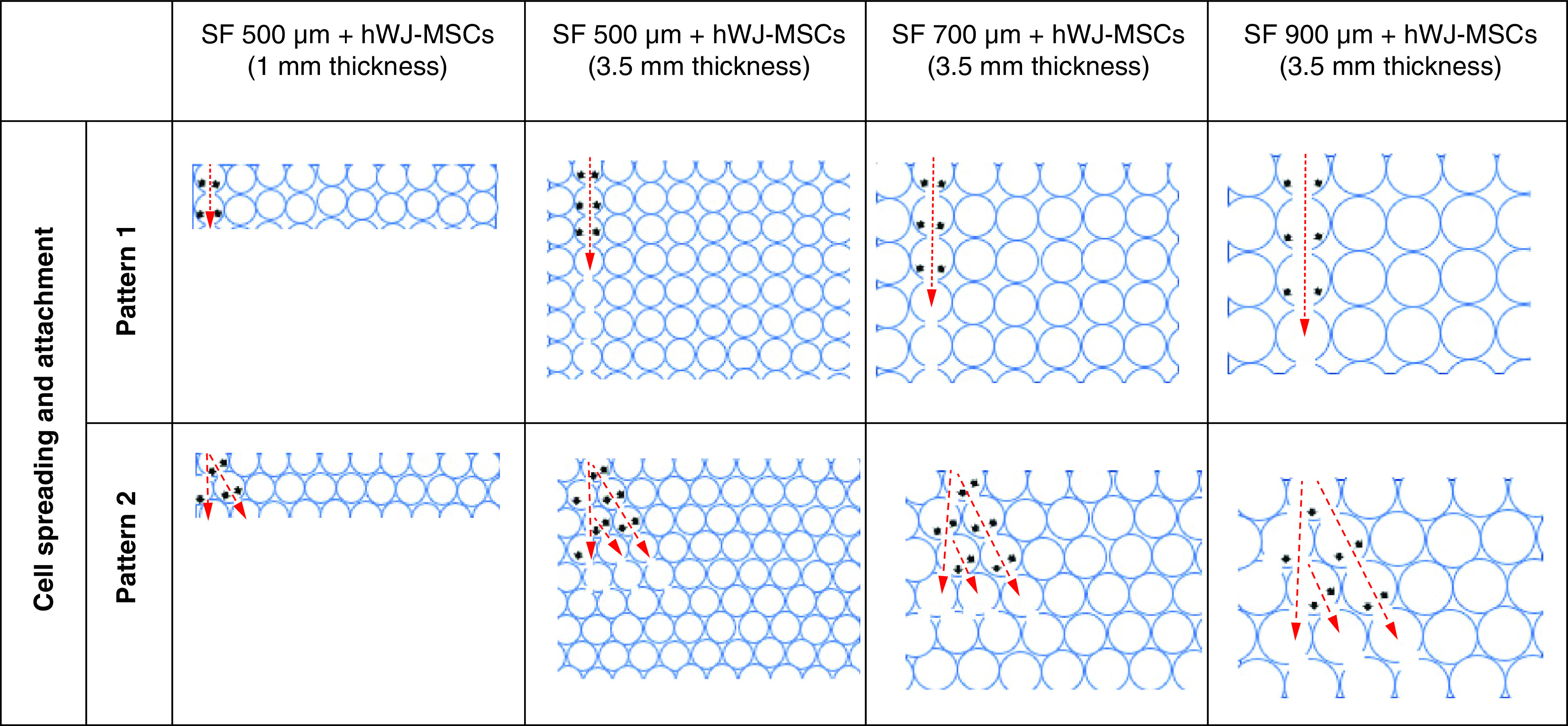
Hypothesis diagram for hWJ-MSC attachment on silk fibroin scaffolds of varying thickness and pore size. The red arrow indicates the direction in which the hWJ-MSC migrated after seeding in one of the pores. The black arrows show possible sites for cell attachment.

Functioning scaffold must include topography, such as related pore structure, that is capable of supporting cell survival, migration and growth nutrition [33]. We discovered that SF scaffold with 500 μm pore size and 3.5 mm thickness is optimal for cell attachment, proliferation and differentiation. Thicker scaffold attaches more cells, but the pore size is also critical for development and growth. This difference occurs because the larger the scaffold pore, the larger the interconnecting pore, making it easier for cells to enter the pore hypothesis in Figure 8. This hypothesis diagram illustrates the relationship between pore size and scaffold height and is corroborated by the data in Figure 2B for adhering cells more highly to the 3.5 mm thick scaffold due to the high number of pores. Hypothesis also corroborated with Figure 3C where thicker scaffold attaches more cells, but the pore size is also critical for development and growth. This assumption is also because larger pores increase the size of the interconnected pore (Table 1), As a result, bigger pores limit cell seeding in one pore, inhibit both proliferation and differentiation.

Water uptake analysis shows that the bigger the pores in the SF scaffold, the higher the effectiveness to absorb the surrounding media, within 1 h (Table 2). Water absorption ranges between 84 and 99%, which is a relatively high proportion for tissue engineering using SF scaffolds manufactured by the salt leaching technique [8,34]. According to Yan *et al.* in [35], the scaffold's porosity also affects the water-binding ability since increasing the porosity improves the water uptake ratio.

The pore size of 500 μm had the highest compression modulus and strength values compared with other pore sizes. The compression modulus findings indicate that a pore with a diameter of 500 μm has the highest number of scaffold stiffness in resisting compressive forces based on surface area. Furthermore, compression strength means that the material's ability with a diameter pore size of 500 μm can maintain the scaffold under stress better than bigger pore sizes. The result of compression modulus related to surface area in 3D SF scaffold is confirmed by Judawisastra, Nugraha and Wibowo in [29] research with the same source and fabricated method of scaffold, who demonstrated that increasing the pore size reduces the surface area with CT analysis.

The compression modulus value in this research using 500 μm diameter pore size is equivalent to other scaffold designs for cartilage tissue engineering (ranging from 0.005 to 5.9 Mpa) [36], but the compressive strength of the SF scaffold is still lower than that of other scaffolds constructions (ranging from 5.27 to 85 Mpa). The compressive test of a porous scaffold is affected by the composition of the material, the pore diameter and the porosity [37]. Additionally, the variation in time between the compression test before and after cell implantation affects compressive strength, particularly in tissue engineering applications [38,39]. In this study, the compression test was conducted solely to determine the strength of the scaffold based on pore size; additional cells were not included. Therefore, for NP implantation, more research needs to be done to find out how cell density affects the modulus test in tissue engineering application.

In SEM analysis (Figure 3A), pores and interconnected pores formed in all variants of the scaffold, regardless of the size of the NaCl used as a porogen (Table 1). The bigger the size of NaCl, the more porous and interconnected pores formed on the scaffold [29]. A functioning scaffold must include a related pore structure capable of supporting cell survival, migration and growth nutrition [33]. Furthermore, the porous linked structure demonstrates physiological conditions similar to those observed *in vivo* [40].

The biocompatibility analysis of hWJ-MSC grown on SF scaffold was tested using the SEM method. Results indicated that cells were able to attach to the SF scaffold by showing the formation of a cell population (Figure 3A) in all the SF scaffold's pore variations. As the primary material for tissue engineering, scaffold SF is known to have biocompatibility properties or the ability to support the attachment and proliferation of hWJ-MSC cells, as demonstrated by Wibowo *et al.* making it ideal for tissue engineering applications [8]. As previously stated Acharya *et al.* in [41], the rough surface of the scaffold is critical for cell adhesion. Biocompatibility is an essential element for the effective use of tissue engineering scaffolds. It enables cells to adhere to the scaffold surface and move while proliferating and differentiating among scaffolds [42].

The effectiveness of cell attachment was evaluated to determine if pore size had an effect during initial cell seeding. In Figure 3B, 500 μm scaffold pore size shows a greater number of percentage cells adhering to the scaffold compared with the larger pore size. These findings shows that 500 μm pore size of SF scaffold proved the highest growth curve and accumulation of GAGs content as differentiation marker until day 21 (Figure 3C–D). The thicker scaffold attaches more cells, but the pore size is also critical for development and growth, because larger pore size of more than 500 μm proves that fewer cells will attach, resulting in decreased cell proliferation and differentiation. The difference in the pore size of the SF scaffold influences the percentage of hWJ-MSC attachment on the scaffold. This difference occurs because the more significant the scaffold pore, the larger the interconnecting pore (Table 1), allowing cells to enter the pore readily. This study corroborates these findings, demonstrating that the maximum growth and differentiation potential was observed at 500 μm pores in studies done by Barlian *et al.* [21].

It is well established that scaffold topography determined by pore size directly impacts the time required for cells to fill the pore region (pore filling) and affects the proliferation of ECM as a sign of chondrocyte differentiation [43]. Additionally, pore size directly affects the time required to fill all pore regions and, therefore, the ECM expression [6]. Further, this investigation established the presence of cell penetration into the SF scaffold across all pore size factors, consistent with Amsar *et al. * [44]. Thus, the SF scaffold with a pore size of 500 μm and a height of 3.5 mm may be capable of supporting hWJ-MSC growth and differentiation. This result is consistent with the previous study [21,45,46] by showing aggrecan and type II collagen as ECM indicators. This result is consistent with the compression test findings (Table 3), which indicate that the pore size of 500 μm had the highest compression modulus and strength values compared with other pore sizes. It is believed that smaller pores have a greater surface area that important for the strength of scaffold [29].

Controlling the differentiation of hWJ-MSC is a critical step following the establishment of cell attachment and proliferation seeded on an SF scaffold for tissue engineering [47]. The addition of 10% PRP increased the differentiating capacity of hWJ-MSC via the secretion of growth factor TGF- β1. These findings are similar to Rosadi *et al.* work on hADSCs [24]. Increasing TGF-β molecules that regulates expression of Sox9 protein via Smad mediated -pathway. Sox9 known as a key transcription factor for chondrogenesis with cartilage matrix expression in extracellular matrix like type II collagen, aggrecan and GAGs. Microenvironment conditioning of low glucose medium has a significant effect on chondrogenesis differentiation, which induces expression of the Sox9 and Col2aI proteins that are involved in chondrogenesis [48]. Meanwhile, examination under hypoxic conditions decreased proliferation and chondrogenic marker compared with normoxic conditions. This result is supported by Naqvi *et al.* using BMSC in [17]. There are two different results regarding how hypoxia affects the growth of hWJ-MSC in monolayer culture conditions. In some studies, there was an increase in growth [49,50], while others demonstrated no significant difference in cell growth grown under normoxia and 5% hypoxia conditions [51,52]. The inconsistency in results is likely due to treatment variations during hWJ-MSC expansion in normoxia or hypoxia, since hWJ-MSC cells are sensitive to hypoxic conditions during the cell expansion phase before treatment.

Given the unique microenvironment circumstances seen in NP, the results of this study show that low glucose concentration is a factor that promotes cell differentiation and may be administered at the start of the cell treatment process and may be employed as an adaptation step before implantation into NP. Low-glucose culture media can simulate low-glucose NP conditions [53]. Despite the low glucose concentrations, cells survived, grew and differentiated by demonstrating ECM chondrogenicity, indicating that low glucose conditions may be employed as an adaptation step before implantation into NP. In contrast, hypoxia becomes a necessary condition that impairs the survival and biological activity of hWJ-MSCs that develop into NP-like cells. Hypoxia could not be given immediately at the start of the procedure to induce differentiation of hWJ-MSC presumably due to an increase in glucose uptake in MSCs during hypoxia as the adaptation process of oxygen changes [54]. Therefore low glucose condition during treatment and hypoxia proved that cells do not receive sufficient glucose sources for metabolic activity and this inhibits cell development [55]. Additionally, culture on 3D scaffold dimension 5 × 5 × 3.5 mm has the impact of lowering the oxygen concentration in the scaffold's core area under hypoxic conditions [56]. This work demonstrated that the SF scaffold architecture and the combination of medium-low glucose and 10% PRP induced chondrogenic differentiation and enhanced proliferation up to day 21 by expressing NP markers such as GAGs content and typre II collagen. These findings demonstrate the originality of the method employing hWJ-MSC as a cell source and the primary form of 3D culture in developing the NP tissue engineering method, which was validated using hypoxic conditions, which mimic the microenvironment in NPs.

The examination under hypoxia conditions decreased proliferation and chondrogenic marker compared with normoxia conditions. Hypoxia, on the other hand, becomes a crucial condition that affects the survival and biological activity of hWJ-MSCs that develop into NP-like cells. Hypoxia could not be given immediately at the start of the procedure to induce differentiation of hWJ-MSC presumably due to an increase in glucose uptake in MSCs during hypoxia as the adaptation process of oxygen changes [54]. Therefore low glucose condition during treatment and hypoxia proved that cells do not receive sufficient glucose sources for metabolic activity and this inhibits cell development [55]. Additionally, 3D culture on scaffold has the impact of lowering the oxygen concentration in the scaffold's core area under hypoxia conditions [56].

In comparison to Mesenchymal Stem Cells, NP cells cultured *in vivo* demonstrated a reduced glucose consumption rate due to an adaptation process to fluctuations in oxygen availability [17]. Thus, it is anticipated that in NP tissue engineering employing hWJ-MSC, cells must be differentiated before being exposed to hypoxic conditions to allow for adaptation to fluctuations in oxygen levels, particularly in IVD or cartilage, which are avascular tissues [57]. This work demonstrated that the SF scaffold architecture and the combination of medium-low glucose and 10% PRP induced chondrogenic differentiation and enhanced proliferation up to day 21 by expressing NP markers such as GAGs content and type II collagen. These findings demonstrate the originality of the method employing hWJ-MSC as a cell source and the primary form of 3D culture in developing the NP tissue engineering method, which was validated using hypoxic conditions, which mimic the microenvironment in NPs.

**Figure 9.**
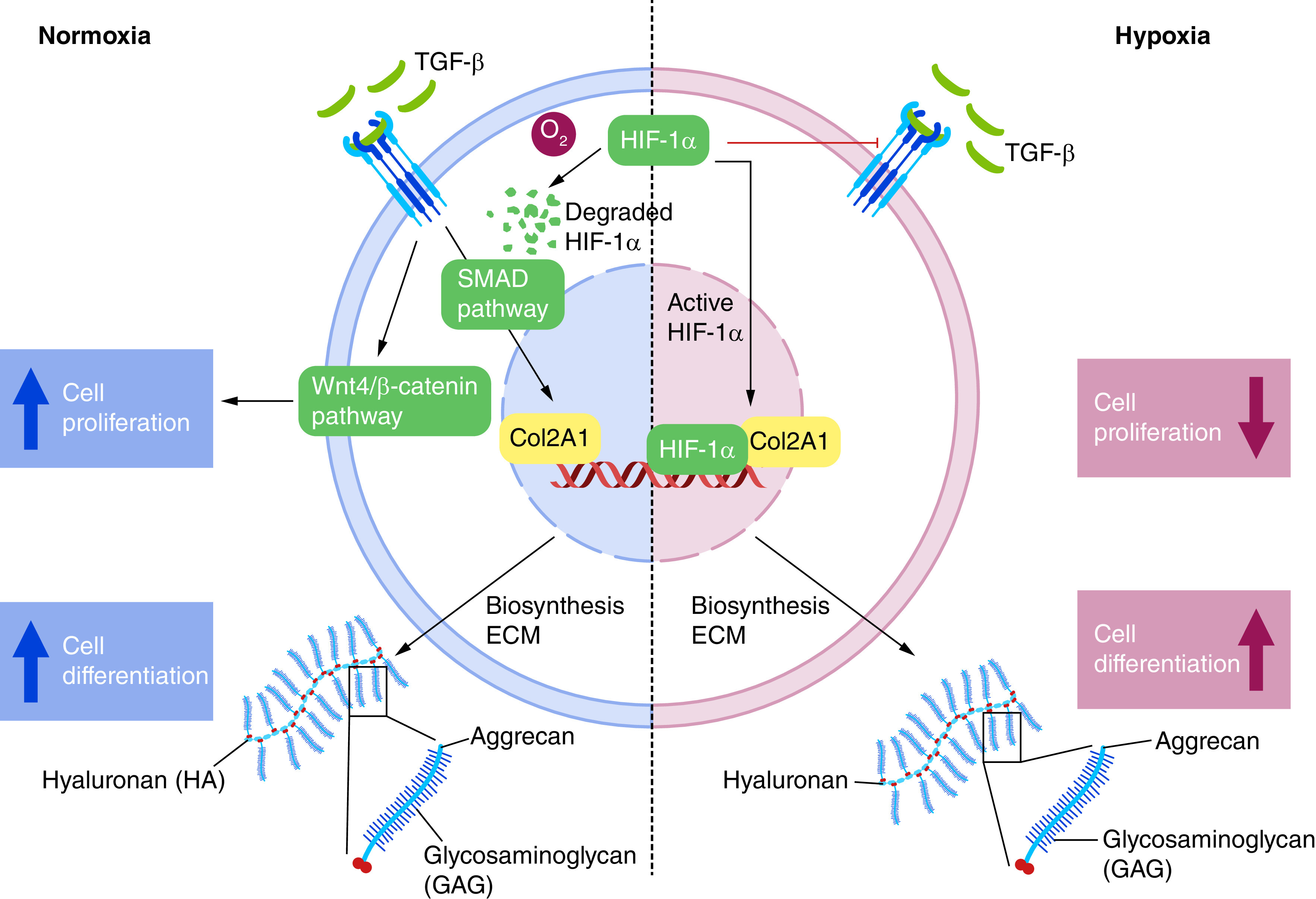
Hypothetical diagram of the role of hypoxia condition. Red arrow means in hypoxia condition, Blue arrow in normoxia condition.

The mechanism behind under normoxia and hypoxia was proposed in Figure 9 and expression of protein related to proliferation and differentiation process shows in Figure 7E & F. In normoxia, culture medium supplemented with 10% PRP increases TGF-β1 molecules induces phosphorylation of the Smad 2/3 effector protein and subsequently translocate into the nucleus to control the expression of genes regulated by Sox 9 (type II collagen, aggrecan and GAG*s*) [58]. TGF-β1 also induces the mechanism of cell proliferation by WNT/β1 catenin pathway [59]. HIF-1α will be degraded by oxygen due to proteasome [60]. In hypoxia condition, there is activated HIF-1α that will work as a Sox9 transcription factor for chondrogenesis by controlling the expression of cartilage matrix components in the ECM [61]. HIF-1α, on the other hand, has antagonist activity with smad 2 and WNT/β catenin pathway. Because hypoxia cannot be employed for stabilization in applying NP tissue engineering utilizing hWJ-MSC, further research may focus on pre-differentiation before cell implantation in NPs, allowing cells to survive and differentiated before being exposed to hypoxic conditions as Wang *et al.* have done [53].

## Conclusion

Scaffold SF with 500 μm pore size and 3.5 mm thickness in combination with low glucose medium supplemented with 10% PRP may stimulate proliferation and differentiation of hWJ-MSCs, resulting in NP-like cells. 3D culture with this combination revealed an increase GAGs content as a marker in NP-phenotype cells and the presence of type II collagen. Because hypoxia cannot be employed for stabilization in applying NP tissue engineering utilizing hWJ-MSC under *in vitro* conditions, further study is required to construct pre-conditions to determine the optimal period for degenerated NPs implantation.

## Future perspective

The current challenge in NP tissue engineering is determining its ideal components such as cell source, the biomaterial and bioactive factor that can sustain survival and function under harsh microenvironment NP conditions, including hypoxia, low oxygen, low pH and hyperosmolarity. The combination of NP tissue engineering in this research: hWJ-MSC as a promising cell source, silk fibroin scaffold as a biomaterial with the best topography and culture conditions interaction may serve as the foundation for future advancements in NP tissue engineering. It also provides clues to the best condition in development of tissue engineering and stem cell therapy, also prepares the next challenge for NP harsh microenvironment condition

**Summary points**

- Combination of silk fibroin scaffold as a biomaterial with the best topography and culture conditions interaction in human wharton's jelly mesenchymal stem cells (hWJ-MSC) growth and differentiation to NP-like cells were observed.
- Growth analysis was performed using MTT assay method and differentiation analysis was performed using GAGs content absorbance method with a spectrophotometer.
- SF scaffold with a minimum thickness 3.5 mm and pore diameter of 500 μm induced the growth and differentiation of hWJ-MSCs.
- *In vitro* culture condition with low glucose medium supplemented with 10% PRP, in hypoxia condition may stimulate proliferation and differentiation of hWJ-MSCs, resulting in NP-like cells.
- Confocal microscopy analysis show that indicated by the accumulation of glycosaminoglycan content and the presence of type II collagen, as markers of NP-like cells.
