# Supplementary material for: The combinatory effect of scaffold topography and culture condition: an approach to nucleus pulposus tissue engineering
Source: Future Sci OA. 2022 Oct 3;8(7):FSO810. doi: 10.2144/fsoa-2021-0157 (PMC9540240; doi:10.2144/fsoa-2021-0157)
Supplement: Supplementary file 1 [file fsoa-08-810-s1.docx]

**Supplementary legend**


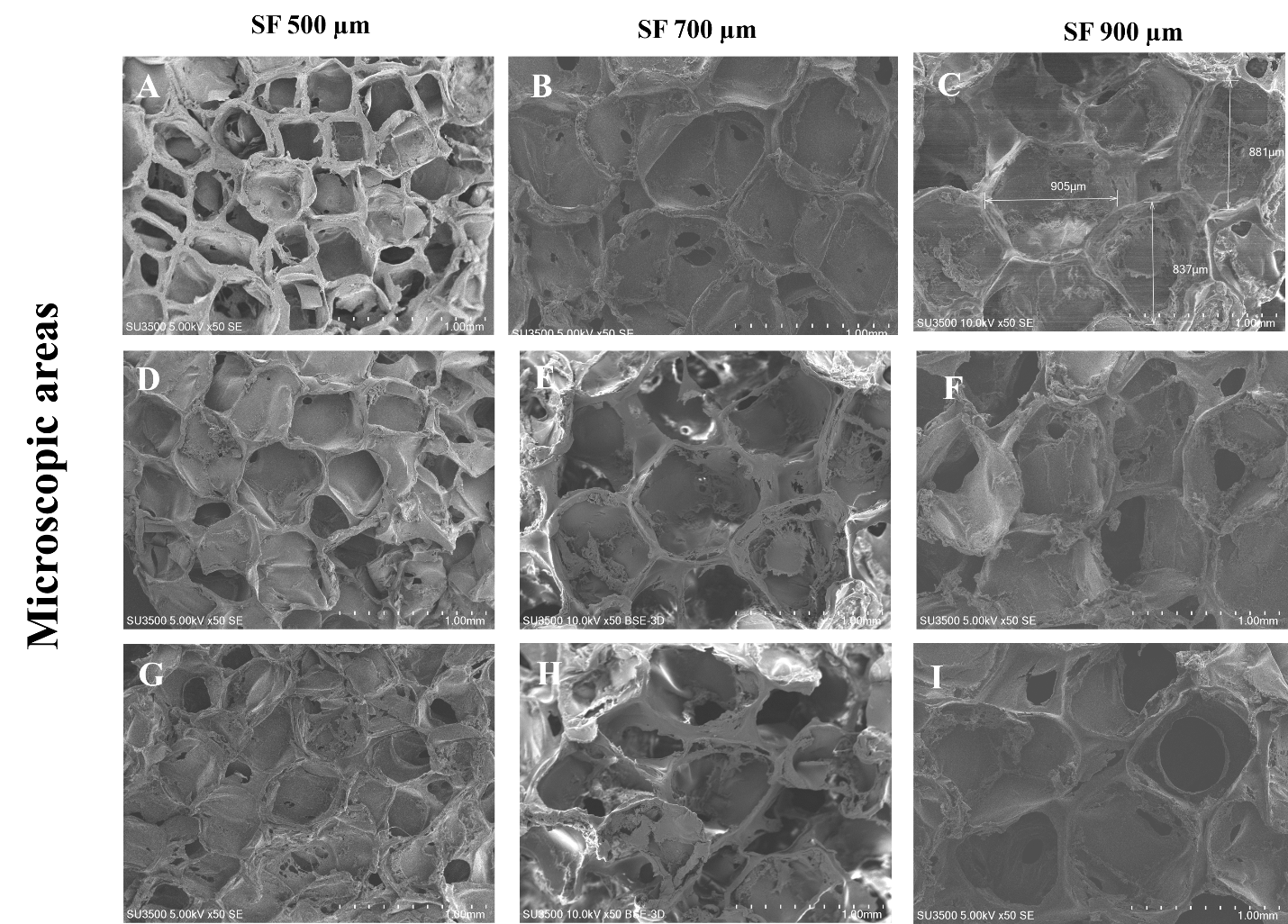


**Supplementary 1**. **SEM observation of SF pore structure in 3 microscopic areas.**
